# Posterolateral Idiopathic Spinal Cord Herniation With CSF Leak in a Pediatric Patient: A Case Report

**DOI:** 10.7759/cureus.96429

**Published:** 2025-11-09

**Authors:** Jordan M Rasmussen, Patrick Opperman, Afshin Salehi, Nicholas Borg

**Affiliations:** 1 Neurosurgery, University of Nebraska Medical Center, Omaha, USA

**Keywords:** dural defect, idiopathic spinal cord herniation, intrathecal fluorescein, pediatric, thoracic myelopathy

## Abstract

Idiopathic spinal cord herniation (ISCH) is a rare cause of thoracic myelopathy, typically presenting in middle-aged adults and involving ventral or ventrolateral displacement of the spinal cord. Pediatric cases and posterolateral dural defects are exceedingly uncommon and may be mistaken for dorsal arachnoid cysts or other mass lesions. We report the case of a 14-year-old girl who presented with progressive thoracic myelopathy following two prior thoracic laminectomies for a presumed arachnoid cyst, neither of which identified a dural defect. Repeat imaging revealed a posterior left dural defect at T8 with focal cord herniation and dorsal CSF collection. Definitive surgical repair was performed using intrathecal fluorescein for localization, microsurgical reduction of the herniated cord, and primary dural closure. Postoperative imaging confirmed resolution of the herniation and CSF collection. The patient experienced full neurological recovery. This case highlights the importance of considering ISCH in pediatric patients with atypical thoracic pathology, particularly when dorsal CSF collections are present without a discrete cyst wall. It also demonstrates the utility of intrathecal fluorescein in reoperative localization of small dural defects and supports direct reduction with primary closure as a definitive treatment approach.

## Introduction

Idiopathic spinal cord herniation (ISCH) is a rare clinical entity, characterized by protrusion of the spinal cord through a dural defect in the absence of trauma, prior surgery, or other secondary causes. First described in 1974 by Wortzman et al. [1], fewer than 200 cases have since been reported in the literature. ISCH typically presents in middle-aged adults and most often involves ventral or ventrolateral herniation at mid-thoracic levels [2-4]. A recent review of published case reports identified posterior or posterolateral herniations in only 1.3% of cases, underscoring the exceptional rarity of this variant [5]. Pediatric presentations are particularly rare, and herniation through a posterior or posterolateral dural defect is even less common [2,3].

The diagnosis of ISCH can be challenging due to its insidious presentation and radiographic overlap with other intradural pathologies. Patients may present with progressive thoracic myelopathy, often with features of Brown-Séquard syndrome. Lesions may be misinterpreted on imaging as arachnoid cysts, spinal cord atrophy, syringomyelia, or other mass-occupying lesions [2,6-8]. These diagnostic ambiguities can lead to delays in treatment or to surgeries that fail to address the underlying pathology.

MRI is the primary diagnostic modality for ISCH and typically reveals focal anterior cord displacement, kinking or “C-shaped” deformation, and obliteration of the anterior subarachnoid space. CT myelography can help define the dural margins and CSF flow abnormalities [3,6]. However, when herniation occurs posteriorly, especially in pediatric patients, radiographic interpretation is more difficult because these defects often show preserved anterior CSF spaces and dorsal fluid collections rather than anterior cord displacement. Surgical reduction of the herniated cord with closure of the dural defect is the standard treatment, typically resulting in clinical stabilization or improvement.

We report a case of posterolateral ISCH in a pediatric patient previously misdiagnosed with arachnoid cysts, who underwent two prior decompressions without identification of a dural defect. We describe the surgical repair, including the use of intrathecal fluorescein to localize the defect, and provide a postoperative outcome supported by imaging and clinical recovery.

## Case presentation

A 14-year-old girl presented with progressive lower extremity weakness, gait instability, and urinary urgency over a two-year period. She had previously undergone thoracic decompressions at outside hospitals-T6-7 and T8-9 laminectomies for a presumed dorsal arachnoid cyst. Neither operation identified a dural defect. She experienced transient symptom relief following each procedure, but her symptoms recurred and gradually worsened. Neurological exam revealed signs of an incomplete Brown-Séquard syndrome, including increased tone and hyperreflexia predominantly on the left side. Strength and sensation were preserved in both lower extremities. Clonus was present bilaterally and more prominent on the left, and Babinski signs were observed bilaterally.

**Figure 1:**
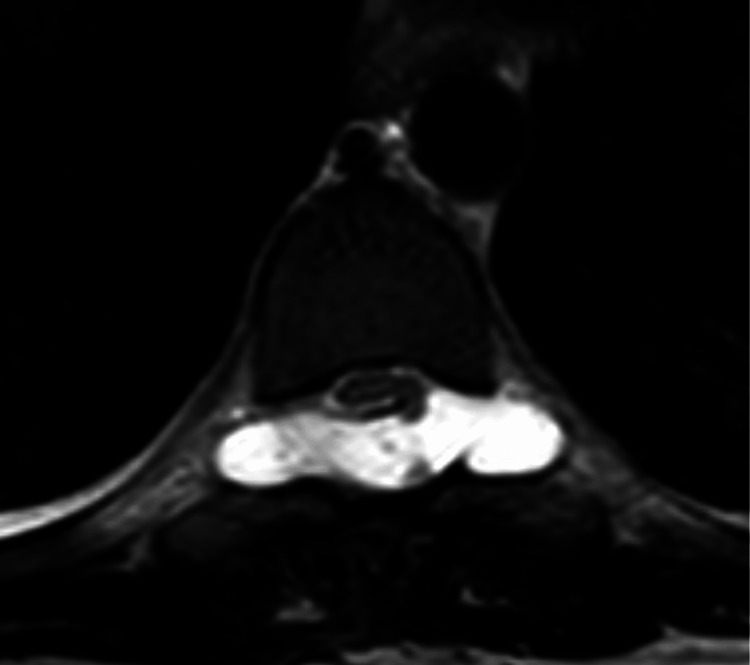
Axial T2-weighted MRI at the T8 level demonstrating a posterior left dural defect with focal spinal cord herniation.

**Figure 2:**
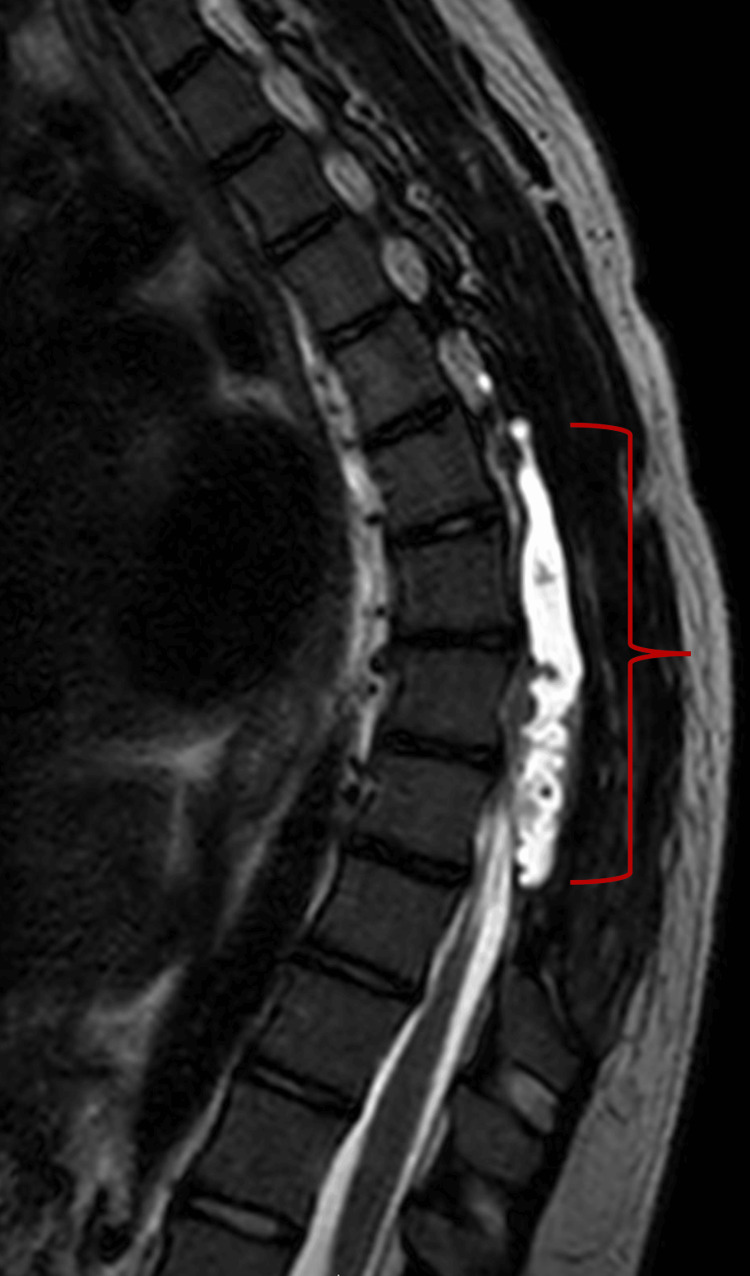
Sagittal T2-weighted MRI of the thoracic spine showing a posterior left dural defect with persistent spinal CSF collection and mass effect on the thoracic spinal cord.

Repeat MRI obtained at our institution revealed a posterior left dural defect at the T8 level with focal herniation of the spinal cord and a persistent dorsal CSF collection exerting mass effect on the thoracic cord (Figures 1, 2). No discrete arachnoid cyst wall or septations were seen, and the spinal cord appeared distorted and displaced. These findings raised concern for ISCH rather than a cystic lesion.

The patient was taken for re-exploration through a T7-T9 laminectomy. Dense epidural fibrosis from prior surgeries was encountered. Upon initial inspection, no obvious dural defect was visible. To aid localization, intrathecal fluorescein was administered via lumbar puncture. After allowing time for rostral migration, a fluorescein-stained area was noted on the dorsal left aspect of the dura at T8, consistent with the site of herniation. The dura was opened in a curvilinear fashion. A 3-mm posterior left dural defect was identified, through which the spinal cord was herniated and adherent to the surrounding dura. Sharp microsurgical dissection was used to release adhesions around the herniated cord, and the spinal cord was gently reduced into the intradural compartment (Video 1). The dural edges were then reapproximated and closed primarily with interrupted 6-0 Prolene sutures without the need for patch augmentation. No CSF leak was observed following closure. The wound was closed in standard multilayer fashion, and no neuromonitoring changes were noted throughout the case. A portion of the capsule wall was submitted for histopathological analysis as part of comprehensive intraoperative evaluation; no fibrous cyst wall was identified, consistent with a CSF collection rather than a true arachnoid cyst.

**Video 1:** Intraoperative repair of idiopathic spinal cord herniation using intrathecal fluorescein. Intraoperative footage demonstrating reoperative exposure, identification of the herniated spinal cord, fluorescein-confirmed CSF egress, microsurgical reduction, and primary dural closure.

**Figure 3:**
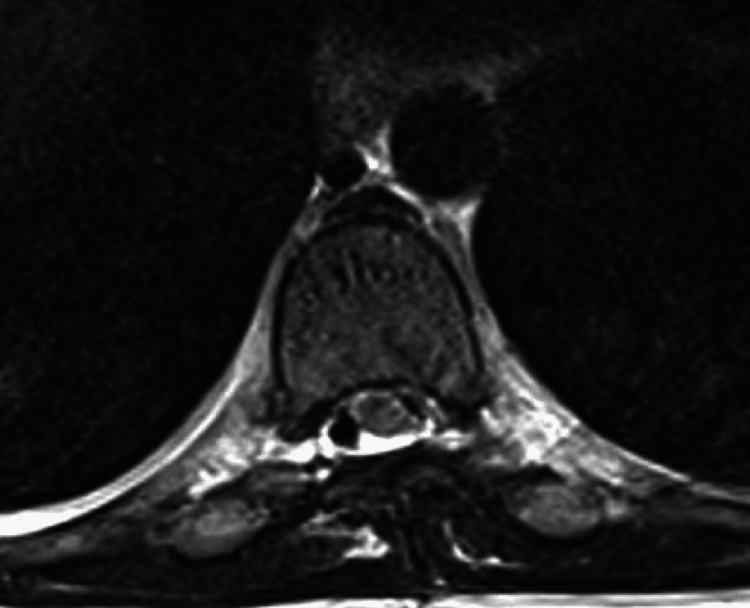
Postoperative axial T2-weighted MRI at the T8 level confirming resolution of the spinal cord herniation without additional or persistent dural defects or epidural fluid collections.

**Figure 4:**
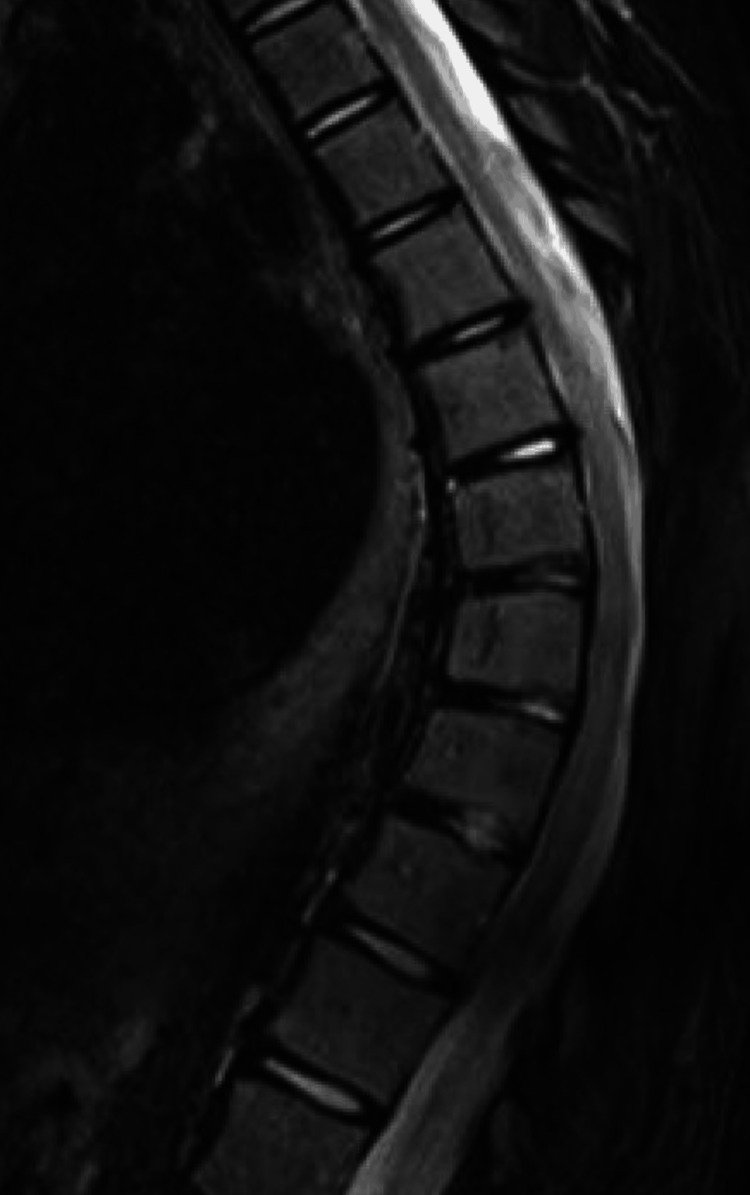
Postoperative sagittal T2-weighted MRI of the thoracic spine showing resolution of the herniation and absence of persistent dural defects or epidural fluid collections. The previously swollen segment of the cord demonstrates normalization.

Postoperative MRI confirmed successful reduction of the spinal cord herniation, with resolution of the dorsal CSF collection and no residual dural defect (Figures 3, 4). The patient’s gait instability and urinary symptoms resolved, and at the three-month follow-up, she had returned to full activity without neurological deficit.

## Discussion

Although limited by a short clinical follow-up period and the absence of detailed quantitative neurological assessment or comparative outcome measures, this case provides important insights into the diagnosis and management of atypical ISCH in the pediatric population. ISCH most commonly presents in middle-aged adults and involves ventral herniation at the T4-T8 levels. Posterior or posterolateral defects, particularly in children, are exceedingly uncommon and can present with atypical imaging findings that mimic other pathologies such as arachnoid cysts [2,6,7].

The underlying pathogenesis of ISCH remains uncertain. Hypotheses include congenital dural weakness, unrecognized trauma, or pressure erosion from adjacent vascular or CSF pulsations. In our patient, the absence of any preceding trauma, infection, or connective tissue disorder supports an idiopathic etiology. It is possible that the dural defect was present at the time of the initial surgeries and went unrecognized, leading to persistent or worsening herniation over time [3,8]. Alternatively, iatrogenic weakening or exacerbation of a dural defect during prior decompressions may have contributed.

Imaging interpretation in ISCH can be challenging, especially in the setting of posterior herniation. When anterior or lateral herniation occurs, the characteristic “C-shaped” deformation of the cord and absence of the ventral CSF space can suggest the diagnosis. However, in posterior herniation, dorsal fluid collections may be misinterpreted as arachnoid cysts, as was the case here. In true arachnoid cysts, a well-demarcated cyst wall is typically present, often with septations. The lack of such findings and the presence of cord tethering or kinking should raise suspicion for an alternative diagnosis such as ISCH [6,7,9].

Multiple surgical strategies have been described for ISCH, including primary dural closure, defect enlargement to prevent retethering, and patch augmentation. The choice of technique depends on defect location, size, and surgeon preference. In our case, primary closure was achieved without difficulty and resulted in complete radiographic and clinical resolution [7,8]. Intraoperative localization of small dural defects, especially in reoperative fields with scarring, can be difficult. Intrathecal fluorescein proved invaluable in this case, offering clear intraoperative visualization of the dural defect. Its use was particularly well suited to a reoperative field with prior surgical scarring, where direct confirmation of CSF egress was essential. Compared to alternatives such as intraoperative ultrasound or endoscopy, fluorescein provided a more immediate and reliable method for defect localization. Its utility has been demonstrated in both cranial and spinal applications and may support broader use in the surgical management of occult dural defects [10-12]. 

## Conclusions

ISCH is a rare but important diagnostic consideration in pediatric patients presenting with progressive thoracic myelopathy and dorsal CSF collections without a clearly defined cyst wall. Early suspicion and careful MRI interpretation are critical to avoid misdiagnosis and repeated ineffective surgeries. This case demonstrates the clinical and radiographic features of posterolateral ISCH, the role of intrathecal fluorescein in defect localization during reoperation, and the effectiveness of primary dural closure for definitive treatment. Although limited by a short follow-up period and single-patient data, this report adds to the growing recognition of atypical ISCH and highlights the need for further studies to guide diagnosis and management in pediatric populations.
